# Key role of MIF-related neuroinflammation in neurodegeneration and cognitive impairment in Alzheimer’s disease

**DOI:** 10.1186/s10020-020-00163-5

**Published:** 2020-04-17

**Authors:** Elham Nasiri, Roman Sankowski, Henriette Dietrich, Aikaterini Oikonomidi, Patricio T. Huerta, Julius Popp, Yousef Al-Abed, Michael Bacher

**Affiliations:** 1 Institute of Immunology, Philipps University Marburg, Marburg, Germany; 2 Center for Molecular Innovation, Feinstein Institutes for Medical Research, Manhasset, NY USA; 3 Elmezzi Graduate School of Molecular Medicine, Feinstein Institutes for Medical Research, Manhasset, NY USA; 4 Current address: Institute of Neuropathology, Faculty of Medicine, University of Freiburg, Freiburg, Germany; 5 Current address: Berta-Ottenstein-Programme for Clinician Scientists, Faculty of Medicine, University of Freiburg, Freiburg, Germany; 6 Old Age Psychiatry, Department of Psychiatry, University hospital of Lausanne, Lausanne, Switzerland; 7 Laboratory of Immune & Neural Networks, Institute of Molecular Medicine, Feinstein Institutes for Medical Research, Northwell Health, Manhasset, NY USA; 8 Institute of Bioelectronic Medicine, Feinstein Institutes for Medical Research, Northwell Health, Manhasset, NY USA; 9 Department of Molecular Medicine, Zucker School of Medicine at Hofstra/Northwell, Manhasset, NY USA; 10 Centre for Gerontopsychiatric Medicine, Department of Geriatric Psychiatry, University Hospital of Psychiatry Zurich, Zurich, Switzerland

**Keywords:** Macrophage migration inhibitory factor, Neuroinflammation, Alzheimer’s disease, Cerebrospinal fluid, Cognitive impairment, Microglia, Astrocyte, ISO-1

## Abstract

**Background:**

Macrophage Migration Inhibitory Factor (MIF) is a potent proinflammatory cytokine that promotes the production of other immune mediators. MIF is produced by most cell types in the brain including microglia, astrocytes and neurons. Enhanced expression of MIF might contribute to the persistent activation of glial, chronic neuroinflammation and neurodegeneration. Here, we investigated the effect of MIF on inflammatory markers and spatial learning in a mouse model of sporadic AD and on tau pathology in AD patients.

**Methods:**

We examined the effects of MIF deficiency and pharmacological MIF inhibition in vitro and in vivo. In vitro, quantitative PCR and ELISA were used to assess cytokine production of STZ-treated glial cells. In vivo, C57BL/6 mice were subjected to intracerebroventricular streptozotocin injection (3 mg/kg, ICV-STZ). Neuroinflammation and contextual learning performance were assessed using quantitative PCR and fear conditioning, respectively. Pharmacological MIF inhibition was achieved with intraperitoneal injections of ISO-1 (daily, IP, 20 mg/kg in 5% DMSO in 0.9% NaCl) for 4 weeks following ICV-STZ injection. The findings from ISO-1 treated mice were confirmed in MIF knockout C57BL/6. To assess the role of MIF in human AD, cerebrospinal fluid levels of MIF and hyperphosphorylated tau were measured using ELISA.

**Results:**

Administration ICV-STZ resulted in hippocampal dependent cognitive impairment. MIF inhibition with ISO-1 significantly improved the STZ-induced impairment in contextual memory performance, indicating MIF-related inflammation as a major contributor to ICV-STZ-induced memory deficits. Furthermore, inhibition of the MIF resulted in reduced cytokine production in vitro and in vivo.

In human subjects with AD at early clinical stages, cerebrospinal fluid levels of MIF were increased in comparison with age-matched controls, and correlated with biomarkers of tau hyper-phosphorylation and neuronal injury hinting at MIF levels as a potential biomarker for early-stage AD.

**Conclusions:**

The present study indicates the key role of MIF in controlling the chronic cytokine release in neuroinflammation related to tau hyperphosphorylation, neurodegeneration, and clinical manifestations of AD, suggesting the potential of MIF inhibition as therapeutic strategy to slow down neurodegeneration and clinical disease progression.

## Introduction

Alzheimer’s Disease (AD) is an aging-associated disease defined by progressive neurodegeneration, neuroinflammation and the presence of protein aggregates consisting of amyloid β (Aβ) and hyperphosphorylated tau (Selkoe 2001). Existing therapeutic options for AD remain inadequate. While Aβ-centric therapies have largely failed to show clinical efficacy, several immunomodulatory therapeutic approaches have been investigated to target chronic neuroinflammation as a key component of AD pathogenesis (Varvel et al. 2009; Lim et al. 2000; Yan et al. 2003). A rationale for implicating inflammatory diseases in AD etiology has been provided by genomic studies showing associations between AD and polymorphisms in a number of genes involved in immune cell function, such as Apolipoprotein E (APOE), Triggering receptor expressed on myeloid cells 2 (TREM2), CD33 (Jonsson et al. 2013; Guerreiro et al. 2013; Griciuc et al. 2013).

Epidemiological evidence and studies in different mouse models have suggested that blocking chronic inflammation associated with the innate immune response of CNS attenuates AD-like pathology (Bacher et al. 2008; Walker and Lue 2007). Therefore, finding a proper target within this inflammatory cascade is of utmost importance, keeping in mind that and ideal therapeutic strategy should inhibit detrimental aspects of the inflammatory response while leaving the beneficial anti-inflammatory response unaffected.

Expressed by neurons and glia, macrophage migration inhibitory factor (MIF) represents a relevant target for anti-inflammatory therapies due to its central role in inflammation (Hoi et al. 2007). The MIF protein forms a donut-shaped homo-trimer with each monomer consisting of six beta sheets and two antiparallel alpha helices (Sugimoto et al. 1996). Depending on the concentration of the protein, both the MIF monomer and trimer exert biological functions (Mischke et al. 1998). These functions comprise enzymatic, cytokine, and chemokine activities (Lue et al. 2002). In contrast to most proinflammatory cytokines, MIF is stored in vesicles as a preformed mediator (Nishino et al. 1995).

Release of MIF from different cell types is triggered by proinflammatory stimuli with lipopolysaccharide (LPS) (Bernhagen et al. 1993), DNA damage and others (Wang et al. 2016). It acts as an early stage cytokine by initiating the inflammatory response and a mediator to maintain the inflammatory response (Bernhagen et al. 1993; Roger et al. 2016). The increase of MIF as an early-stage cytokine is also associated with the release of other cytokines, contributing to chronic neuroinflammation and possibly accelerating the neurodegenerative process. Initially proinflammarory cytokines activate microglia and subsequently enhance the clearance of detritus from pathological processes. However, prolonged expression of these immune mediators might have detrimental effects in the CNS (Krstic and Knuesel 2013).

MIF’s role in AD pathology has been investigated in many aspects, including immune response, insulin regulation and oxidative stress (Bacher et al. 2010; Kassaar et al. 2017). In AD pathology, MIF mainly binds the CD74/CD44 receptor complex followed by multiple intracellular signaling pathways, such as the activation of the extracellular signal regulated kinase (ERK) 1 and 2, the Phosphoinositid-3-Kinase (PI3K)-Akt signal transduction cascade, Nuclear factor ‘kappa-light-chain-enhancer’ of activated B-cells (NFκB), and the Adenosinmonophosphat (AMP)-activated protein kinase (AMPK) pathway (Su et al. 2017). Of note, a recent report performed on human AD brain samples suggests a causal relationship between certain CD44 splice variants and neuronal cell death, thus indicating that CD44 contributes to AD pathology in humans (Pinner et al. 2017). Furthermore, it has been demonstrated that attenuation of MIF in experimental models of AD dampens the astrocytes activation and tau hyperphosphorylation (Li et al. 2015).

A number of AD mouse models exist. Most transgenic models display overexpression of mutated human Aβ or tau proteins. While these models were crucial in understanding the effects of Aβ and tau proteins on cellular brain function and cognition, clinical studies targeting Aβ and tau have so far failed to show efficacy (Nazem et al. 2015). However, after Biogen’s initial failure in two clinical trials of Aducanumab, reanalysis of the clinical trial data presented during the Clinical Trials in Alzheimer’s Disease conference in December 2019, surprisingly suggested a turnaround by providing information that the highest dose of aducanumab just might slow down the cognitive and functional decline caused by AD.

The ICV-STZ mouse model is a non-transgenic mouse model mimicking some aspects of sporadic AD, including neuroinflammation, disruption of tau phosphorylation, insulin signaling, Aβ pathology and cognitive deficits (Nazem et al. 2015; Grunblatt et al. 2007; Salkovic-Petrisic et al. 2006; Chen et al. 2012; Grieb 2016; Zhang et al. 2018).

Here we investigated the role of MIF as upstream regulator for cytokine production from glia cells during neuroinflammation. It was shown before that MIF deficiency attenuates tau hyperphosphorylation and astrocytic activation (Li et al. 2015). However, the effects of MIF deficiency on proinflammatory cytokines and cognition in vivo in the ICV-STZ model have not yet been addressed. To this end, we have assessed the STZ-induced inflammatory response in vitro and in vivo in ISO-1 treated and in MIF deficient mice. Furthermore, we found a robust correlation between MIF levels and hyperphosphorylated tau in the cerebrospinal fluid (CSF) of AD patients. This is in line with a previous report that stated that MIF-related inflammation is associated to amyloid pathology, tau hyperphosphorylation, and neuronal injury at the early clinical stages of AD (Oikonomidi et al. 2017).

Our findings corroborate a crucial role of MIF in AD pathology and highlight its diagnostic and therapeutic potential.

## Methods

### Primary microglia and astrocytes cell culture

Primary microglia were prepared from mice at postnatal day p3. Using magnetic activated cell sorting technology by MACS® Neural Tissue Dissociation Kit following the manufacturer’s protocol. Astrocytes cells were separated from the forebrains of mouse pups at E16. Cerebellum was removed and sterile scalpel was used to incise down the midline of the brain. Tissues were dissected a culture dish containing 37 °C HBSS. The isolation of the cells was performed in accordance with the Siebenheber and Wooten protocol (Seibenhener and Wooten 2012).

Cells from both sexes were included in the culture. DMEM medium with 10% glucose, 10% fetal bovine serum (FBS, Thermo Fisher Scientific, Waltham, MA, USA) and 1% penicillin-streptomycin (Thermo Fisher Scientific) was used to maintain the cells. The purity of isolated microglia, astrocyte cells was determined by Western blot using anti-Iba1 (Fujifilm Wako Chemicals, Osaka, Japan), GFAP (Cell Signaling Technologies, Frankfurt am Main, Germany) and β-Actin (Cell Signaling) antibodies prior to performing in vitro experiments (Suppl. Figure 1).

Streptozotocin (STZ, Zanosar™ Teva Phramaceuticals, North Wales, PA, USA) was used to for cell treament. Cell viability was determined by XTT assay to determine the working concentration of the STZ for each cell type. Supernatants were collected for ELISA (R&D Systems, Minneapolis, MN, USA) at different time points, and the cells were used for isolation of mRNA using QIAGEN RNeasy mini-kit (Qiagen, Hilden, Germany).

### Animals

Male C57BL/6 (*n* = 20) and male MIF-KO (*n* = 20) mice were used for this study (6 month, Jackson Laboratories, Bar Harbor, ME). MIF KO mice were backcrossed into C57BL/6 for six to eight generations and bred using homozygous MIF KO animals (Jackson Laboratories, Bar Harbor, ME, USA; (Bozza et al. 1999)). Genomic PCR was performed to genotype MIF locus of all progeny. Covariates such as litter size (5 mice per cage), cohort size (*n* = 10 mice tested at a time) and enrichment were kept constant across the tested groups. The mice were kept on a reverse light/dark cycle (9 am to 9 pm: dark) with ad libitum access to chow and water. All experiments were performed at the dark cycle and in accordance with the NIH guideline under approved protocols by animal Committee of the Feinstein Institute for Medical Research, Northwell Health System.

### Intracerebroventricular (ICV) injection of STZ or vehicle

STZ (Zanosar™ Teva Phramaceuticals) or vehicle (phosphate saline buffer used for dissolving STZ) were stereotaxically injected into the left lateral ventricle of 6-month-old male C57BL/6 or MIF-KO mice. The animals were anesthetized using 3% inhalant Isoflurane (Attane™, Minrad Inc., Orchard Park, NY, USA) and positioned into a stereotactic apparatus (David Kopf instruments, Tujunga, CA, USA). Each animal received 3.0 mg/kg STZ in 2.0 ul 0.9% saline into the left ventricle of the brain, using the bregma zero coordinate (place of injection, − 1.0 mm lateral, − 0.3 mm posterior and − 2.5 mm below). For analgesia, animals received buprenex post operatively 0.1 mg/kg subcutaneously.

ISO-1 was synthesized at the Al-Abed lab as previously described (Xue et al. 1997). The ICV-STZ group received either 20 mg/kg ISO-1 in 5% DMSO in 0.9% NaCl or vehicle (5% DMSO in 0.9% NaCl) starting day 3 after surgery. CNS efficacy of ISO-1 has previously been demonstrated (Conboy et al. 2011). Behavioral tests were performed 28 days after the surgery. Brains were removed immediately, the hippocampus was isolated, homogenized in TRIZOL reagent (Thermo Fisher Scientific) and flash frozen in liquid nitrogen followed by storage at − 80 until mRNA isolation.

### Quantitative PCR

The hippocampi of the animals were stored in 200 μl TRIZOL reagent (Thermo Fisher Scientific) for RNA isolation. 50 μl 1-Bromo-3-chloropropan was added to the samples and the mRNA was isolated using QiAgen mRNA isolation kit (Qiagen). 0.5 μg RNA was retrotranscribed using iScript cDNA synthesis kit (Biorad, Hercules, CA, USA). Primers were designed based on accession numbers from a library of primers for SYBR green and were ordered from Fisher Scientific. Quantitative PCR (qPCR) was performed on a LightCycler 480 (Roche Life Science, Basel, CH) using SYBR Green Master Mix (Merck, Darmstadt, Germany). The quantity of target genes was normalized to housekeeping gene of choice (β-actin) using the comparative Threshold Cycle (CT) method (ΔΔCT), and *n* = 10 samples were used for controlling each cohort. In this method, the average of the Ct values for the house-keeping gene and the target genes of interest are compared in the experimental and control conditions, returning 4 different values. ΔΔCT is calculated by subtracting differences between target and housekeeping values under control condition from differences between target and housekeeping values under experimental conditions. Value of 2^ ΔΔCT is calculated to get the expression fold change. Results were expressed as mean ± standard error of the mean (SE) of at least four different animals for each experimental group.

### Behavioral assessment, fear conditioning

Fear conditioning was used for assessing contextual memory in mice. It was performed in a conditioning chamber (clear Plexiglas, dim light, metal grid floor) with a video camera mounted on the top of the chamber for recording the trials. FreezeFrame software (Coulbourn Instruments, Holliston, MA) was used to analyze the videos. Mice were habituated to the chamber on the day 1 for 10 min. On day 2, mice were given five paired conditional stimuli (tone, 20-s long, 5 kHz, 80 dB) co-terminated with an unconditional stimulus (foot shock, 1 s, 1 mA). On day 3, animals were placed back in the chamber and ‘freezing’ of each individual mouse was measured as fear response in the form of total freezing time.

Blood plasma and cerebrospinal fluid MIF levels in patients with AD and controls.

Cerebrospinal fluid (CSF) and plasma MIF levels were measured in subjects with early clinical AD (i.e. mild cognitive impairment (MCI) or mild dementia with core AD pathology confirmed by well-established CSF biomarkers; *N* = 19) and healthy controls with normal cognition and matched for age, gender and education (*N* = 14). As an important proportion of elderly subjects with normal cognition may have cerebral AD pathology thus being at preclinical stages of the disease, we included in the control group only participants with normal CSF AD biomarkers as defined as a CSF ptau 181/Aβ1–42 ratio < 0.0779, as previously described (Popp et al. 2017). All study participants were recruited and assessed at the Department of Psychiatry, University Hospital of Lausanne, Switzerland.

### Ethical statement

All in vivo animal experiments were performed in accordance to the National Institute of Health (NIH) guidelines and under protocols approved by the institutional animal care and use committee (IACUC) of the Feinstein Institute for Medical Research. The institutional ethical committee from the University Hospitals of Lausanne approved the protocol of the human study (No. 171/2013) and all participants signed written informed consent.

### Data analysis

Graphs were prepared using Prism software. Data are expressed as means ± SEM. Statistical analysis was performed using the R program environment or Prism software (Team RC 2013). For fear conditioning, the daily performance of the treatment groups was analyzed using One or Two-way ANOVA followed by paired student T test or Bonferroni’s post-test. Paired student t test was performed on ELISA results to test against the null hypothesis and Tukey test was used to assess differential expression on qPCR data.

To verify whether MIF concentrations differ between subjects with AD and controls we used the Kruskal-Wallis test for group comparison. Continuous variables not normally distributed according to the Kolmogorov-Smirnov test were log-transformed. To further explore whether MIF concentrations may be related to amyloid pathology, neuronal injury, and tau hyperphosphorylation two-sided correlation analyses between the MIF levels and the CSF concentrations of Aβ1–42, tau and ptau181, respectively, were performed using the Pearson’s statistics.

## Results

### Streptozotocin induced extracellular MIF release

**Fig. 1:**
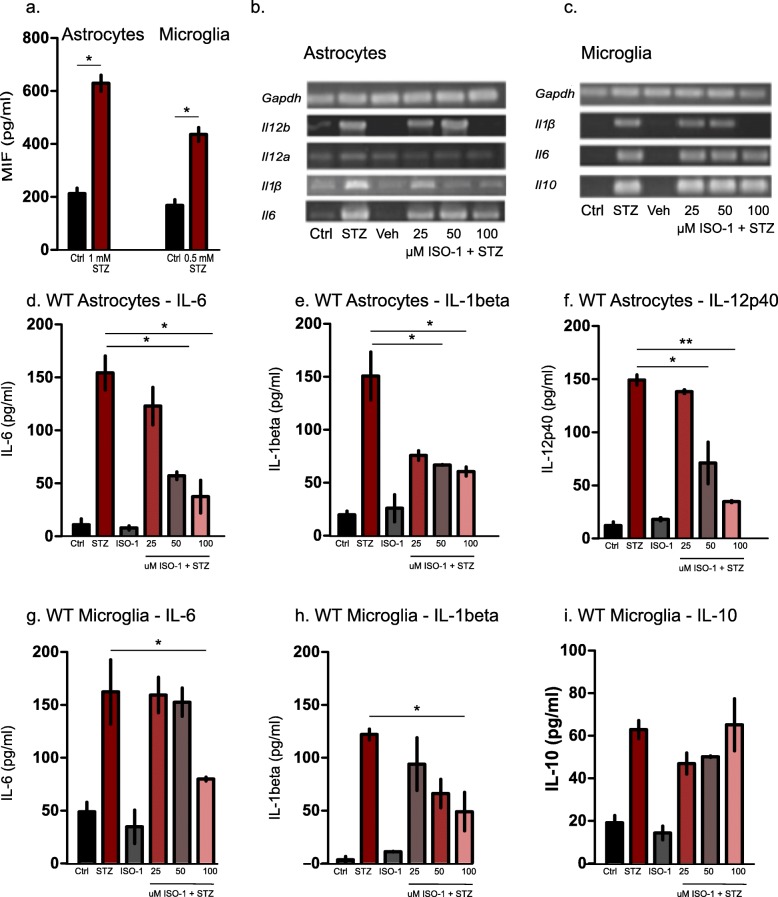
In-vitro results of STZ stimulation on murine Microglia and Astrocytes. **a** ELISA of MIF secretion in supernatants of astrocytes and microglia after 24 h STZ treatment. Graphs represent the mean of *n* = 3 biological replicates (two technical replicates for each) with standard errors of the mean (± SEM), (*P < 0.05, Student’s t test). **b** and **c** mRNA expression levels for different cytokine in response to STZ treatment in astrocytes (**b**) and microglia (**c**) with and without ISO-1 treatment. **d**- **f**. Cytokine ELISA of astrocytes after 24 h STZ treatment. Data are means of *n* = 3 independent biological replicates, error bars represents ± SEM (*P < 0.05, Student’s t test). IL-6 (**d**), IL-1β (**e**) and IL-12p40 (**f**) were released in response to STZ treatment in astrocytes using different concentrations of ISO-1 to inhibit MIF resulted in dose dependent decrease in cytokine release in both cell types. **g-i** Cytokine ELISA in wild type microglia. IL-6 (**g**) IL-1β (**h**) and IL-10 (**i**) were measured after STZ treatment with and without ISO-1. One-way ANOVA with Tukey’s multiple comparison test was performed. (*P < 0.05, **P < 0.01)

Primary cell cultures of astrocytes and microglia from C57BL/6 mice were incubated with 0.5 mM and 1 mM STZ (respectively for astrocytes and microglia). Using ELISA, we observed a significant increase of extracellular MIF levels in both cell types after 24 h (Fig. 1a). It has been previously shown that upregulation of *Mif* transcription is delayed (Lanahan et al. 1992) and that MIF protein is pre-stored intracellularly, which allows for its release as an early-phase cytokine (Atsumi et al. 2007). ISO-1 was previously shown to block the tautomerase active site of MIF molecule without affecting the amount of the protein itself (Al-Abed et al. 2005).

STZ treatment induced MIF-dependent IL-1β and IL-6 production at both transcriptional and translational levels.

Using primary microglia and astrocytes, we assayed for IL-6 and IL-1β cytokine production, at mRNA and protein levels. IL-6 is classically considered a proinflammatory cytokine which was also shown to have regenerative activity (Scheller et al. 2011). MIF regulates *Il6* gene expression by influencing NF-kβ (Chuang et al. 2010). Although astrocytes are known to be the main source of this cytokine (Quintana et al. 2013), microglial expression of *Il6* increases dramatically in the brain of aged mice (Van Wagoner et al. 1999), which is associated with cognitive decline. IL-6 secretion was increased both at RNA expression (Fig. 1b, c) and extracellular protein levels in response to STZ treatment and attenuated by ISO-1 treatment (Fig. 1d, g). IL-12p40 secretion in response to STZ was observed only in astrocytes. It was attenuated in a dose dependent manner in response to ISO-1 (Fig. 1b, f). Gene expression and protein levels of IL-1β secretion were significantly and dose dependently inhibited by the MIF inhibitor ISO-1 (Fig. 1b, c, e, h). Thus, while STZ treatment triggered the secretion of MIF, IL-1β and IL-6, the secretion of the latter two cytokines was attenuated under ISO-1 treatment.

### STZ-induced expression of IL-10 in microglia was not MIF dependent

STZ was shown to induce the release of proinflammatory mediators, such as IL-6 and TNF- α (Sun et al. 2005). To further investigate these findings in our model, we investigated the anti-inflammatory cytokine IL-10 on transcriptional and translational levels (Strle et al. 2001). We found that STZ led to increased IL-10 secretion in microglia, which remained unaffected even at the highest concentration of ISO-1 (100 μM, Fig. 1c, i). Thus, MIF inhibition with ISO-1 had an effect on the extracellular levels of the proinflammatory cytokines IL-6, IL-1β and IL-12p40, but not on the anti-inflammatory cytokine IL-10.

### Pharmacological MIF inhibition did not affect cytokine expression in ICV-STZ model

**Fig. 2:**
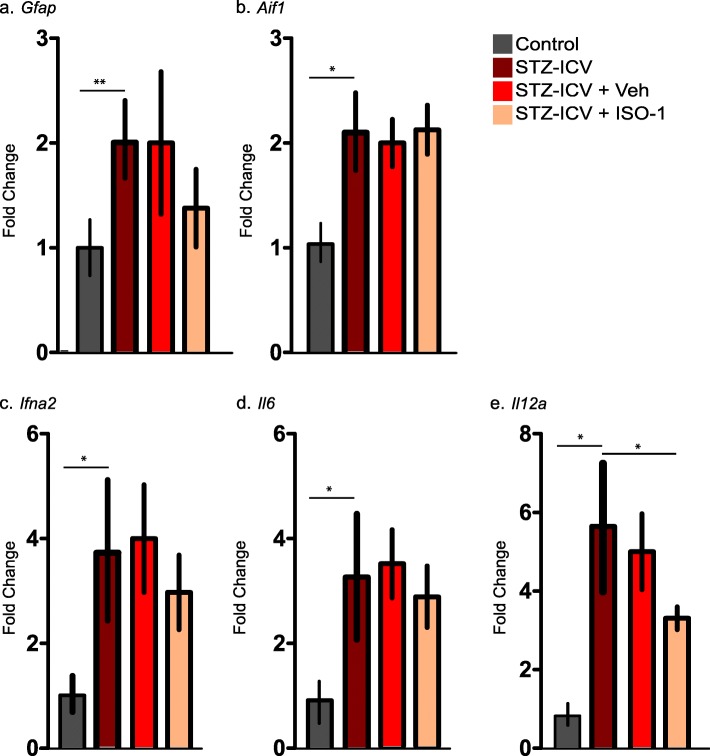
In vivo regulation of GFAP, Iba1 and proinflammatory cytokines in different treatment groups of C57BL6 mice. qPCR of *Gfap* (**a**) and *Aif1* (**b**), *TNF-alpha* (**c**), *Il6* (**d**) and *Il12a* (**e**) ex vivo after hippocampal ICV-STZ. Graphs represent the mean ± SEM of 4 to 6 animals, tested in qPCR and ran as duplicate technical replicates. One-way ANOVA with Tukey’s multiple comparison test was performed. (*P < 0.05)

To investigate the effect of MIF inhibition on cytokine production in the ICV-STZ in vivo model, mRNA was extracted from hippocampi of different experimental groups of mice and reverse-transcribed into cDNA to investigate expression of several inflammatory cytokines. As a first step, we looked for upregulation in *Gfap* and *Aif1* (encoding the protein Iba1) as markers for astrocytes and microglia. We observed a significant increase in both, *Gfap* and *Aif1*, as well as the cytokines *Il6* and *Il12a* (Fig. 2a-e). Expression of these genes was not affected in ISO-1 treated ICV-STZ C57BL/6. However, we observed a downregulation trend in the case of *Gfap*, *Ifna2, Il6 and Il12a.*

### Pharmacological MIF inhibition in ICV-STZ mice influences spatial strategy preference and contextual memory

It has previously been shown that ICV injection of STZ is followed by tissue damage and neurodegeneration in the hippocampus (Kraska et al. 2012), and the inhibition of MIF in ICV-STZ model, attenuated the hyperphosphorylation of tau protein and astrocyte activation (Li et al. 2015). Considering the in vitro data for MIF inhibition resulting in attenuation of cytokine release in both microglia and astrocytes, we were interested in testing hippocampal dependent learning contextual memory using fear conditioning.

Before behavioral testing, we conducted a primary screening to assess confounding effects of ICV-STZ. To this end, mice were tested for muscle and spinal, spino-cerebellar, sensory and autonomic functions. Notably, we found no differences between ICV-STZ and ICV-Veh groups (not shown).

ICV-STZ has been shown to induce contextual memory deficits in mice (Zhang et al. 2018). To assess the involvement of MIF in contextual memory deficits, we tested the behavior of the different experimental groups using the fear conditioning paradigm. In this test, the response to a chamber associated with a frightening experience can be quantified as increased freezing (time spent without moving) by animals that remember this chamber.

**Fig. 3:**
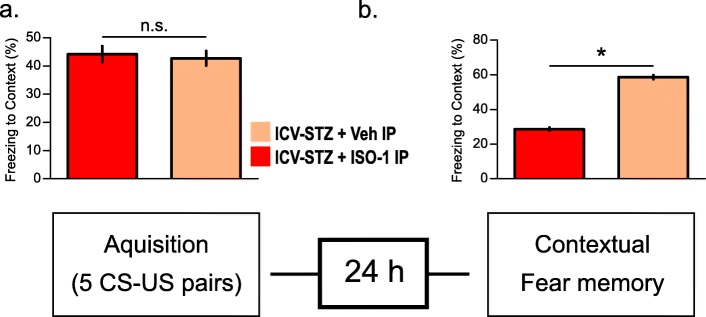
Contextual fear conditioning in ICV-STZ wild type animals when treated with ISO-1 vs Vehicle. **a** Freezing during trace fear-conditioning (training session, *n* = 5 for each experimental group). **b** Freezing 24 h after fear conditioning session. Data is represented as mean ± SEM for *n* = 5 per group. Statistical testing was performed using Student’s t-test (*P < 0.05, n.s. - not significant)

We observed no significant difference between Veh-IP and ISO-1-IP (*N* = 10, daily, IP, 20 mg/kg) treated ICV-STZ mice with respect to the amount of freezing during the acquisition phase (Fig. 3a). This indicated that ISO-1 treatment did not affect the baseline response to the test. Strikingly, ISO-1-IP treated ICV-STZ mice froze significantly more in comparison to IP-Veh ICV-STZ animals, indicating that inhibition of the MIF results in a significant increase in the average freezing time of these animals, representing memory improvement (Fig. 3b). Taken together, pharmacological inhibition of MIF using ISO-1 prevented spatial reference and context learning deficits in the ICV-STZ mouse model.

### MIF is necessary for ICV-STZ induced cytokine production

**Fig. 4:**
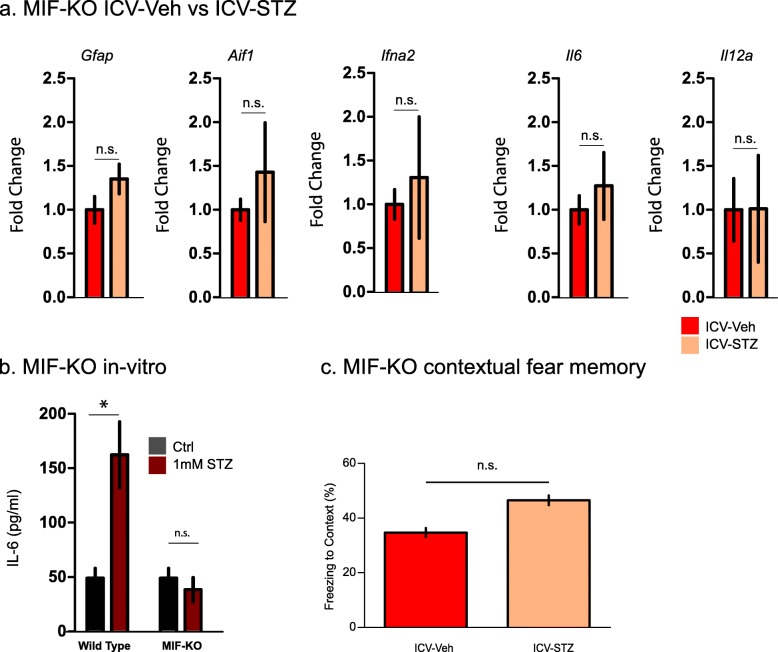
Effects of MIF deficiency in MIF-KO mice and cells, in vitro and ex vivo and in vivo. **a** qPCR off hippocampal cytokine expression in MIF-KO mice after ICV-STZ compared to ICV-Veh treatment. Bars represent the mean ± SEM) of 4 to 6 animals ran as duplicate technical replicates. Statistical testing was performed using Student’s t-test. **b**. IL-6 ELISA in STZ-treated wild type and MIF-KO astrocytes. Data is presented as means ± SEM from *n* = 3 biological replicates (**c**). Freezing in the contextual fear conditioning task of ICV- with respect to ICV-Veh treated STZ MIF-KO mice. Data are presented as mean ± SEM for *n* = 6 animals per group. Statistical testing was performed using Student’s t-test (*P < 0.05, n.s. - not significant)

To control for possible off-target effects of ISO-1, we examined the effect of ICV-STZ injection in MIF-KO mice. Notably, we observed no upregulation in the mRNA levels for glial markers such as *Gfap* and *Aif1* as well as the cytokines *Il6* and *Il12a* in hippocampi of ICV-STZ injected MIF-KO mice in comparison to ICH-Veh, which served as control group (Fig. 4a). Consistently with that, STZ-treated primary astrocytes isolated from MIF-KO mice showed no increase in IL-6 production assessed by ELISA compared to WT primary astrocytes (Fig. 4b). Notably, ICV-STZ and ICV-Veh treated MIF-KO mice showed no differences in average freezing time indicating preservation of contextual memory in the absence of MIF (Fig. 4c). Thus, MIF deficient mice were protected from ICV-STZ induced upregulation of cytokines and context memory deficits.

### Cerebrospinal fluid MIF concentrations are increased in subjects with early AD and correlate with tau and hyperphosphorylated tau levels

**Fig. 5:**
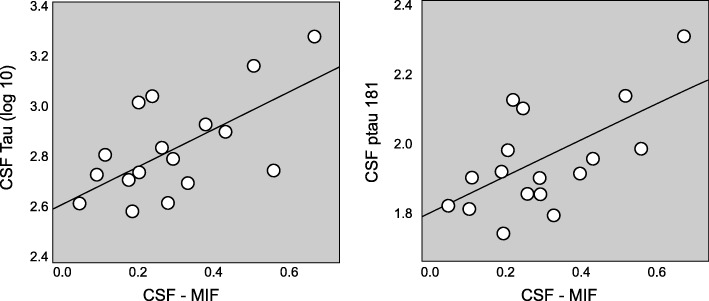
Correlations between the CSF concentrations of MIF and tau. Correlations between the CSF concentrations of MIF and (**a**) tau, and (**b**) ptau181 in subjects with early stage AD. CSF MIF concentrations were correlated with CSF tau and ptau181 levels (log_10_-transformed concentrations) (*r* = 0.629, *p* = 0.004 and *r* = 0.612, *p* = 0.005, respectively). Each dot represents a case from *n* = 19 cases and *n* = 14 controls matched for age, gender and education

Given the previously established involvement of MIF in tau hyperphosphorylation (Li et al. 2015) and our findings that MIF inhibition and deficiency protected from ICV-STZ-induced cytokine induction and spatial learning deficits, we were wondering about the role of MIF in human AD. To this end we analyzed CSF levels of MIF in patients with AD (see Table 1 for demographics and clinical characteristics of the included participants). CSF MIF levels, but not plasma levels were increased at a trend level (*p* = 0.058) in AD subjects compared to the controls (Bacher et al. 2010; Popp et al. 2009). While no correlation has been found between CSF MIF levels and the global cognitive performance as assessed by the Mini Mental State Examination (Folstein et al. 1975) we observed a robust correlation between the CSF levels of MIF with the CSF levels of tau and p-tau 181 (Fig. 5). Notably, we found no correlation between CSF levels of MIF and Aβ1–42 (not shown).

**Table 1:** Clinical characteristics and biomarker measures

	Controls (*n* = 14)	AD (*n* = 19)
Age (years), mean (SD)	70.5 (4.1)	69.7 (4.6)
Gender, No. (%) of males	5 (35.7%)	10 (52.6%)
Education years, mean (SD)	13.1 (2.6)	13.3 (2.5)
MMSE scale, mean (SD)	28.6 (1.8)	23.8 (3.9)*
APOEε4 carriers, No. (%)	2 (14.3%)	9 (47.4%)*
CSF MIF (ng/ml), mean (SD)	0.158 (0.096)	0.270 (0.168)
Plasma MIF (ng/ml), mean (SD)	0.045 (0.077)	0.113 (0.170)
CSF Aβ 1–42 (pg/ml), mean (SD)	990.3 (203.9)	522.8 (134.0)*
CSF tau (pg/ml), mean (SD)	182.1 (44.4)	738.5 (407.9)*
CSF p-tau181 (pg/ml), mean (SD)	42.6 (12.4)	93.9 (35.7)*

*MIF* macrophage migration inhibitory factor, *APOEε4* Apolipoprotein E ε4 allele, *MMSE* Mini Mental State Examination

*statistically different (*p* ≤ 0.05) from CDR 0, using Kruskal-Wallis test for continuous variables and binomial proportion tests for categorical variables

## Discussion

In this study, we explore the role of MIF in neuroinflammation, tau phosphorylation and cognitive deficits in a mouse model of sporadic AD and human subjects with AD. In vitro experiments on primary glia cell cultures suggested a role of MIF in promoting neuroinflammation, by regulating the production of other proinflammatory cytokines. We demonstrated that pharmacological MIF inhibition and MIF deficiency conveyed protection from spatial learning deficits in the ICV-STZ mouse model of sporadic AD. Finally, phosphorylation of tau was positively correlated with MIF levels in AD patients. Our data suggest that MIF inhibition alleviates inflammation by down-regulating production of proinflammatory cytokines, resulting in improvement of cognitive function. Our findings provide a direct link between neuroinflammation, as a recognized causing factor of sporadic AD (Krstic and Knuesel 2013), tau phosphorylation, the most important biomarker for AD progression (Braak and Braak 1991) and cognitive deficits as the defining symptom and major driver of disability in AD (Cummings 2005).

The complex pathology of AD, combined with the clinical failure rate of drugs designed for amyloid reduction, have also raised concerns as to whether targeting amyloid metabolism might be sufficient as a therapeutic approach. The observation that people with rheumatoid arthritis had an unexpectedly low prevalence of dementia indicates that anti-inflammatory drugs might lessen the risk of Alzheimer’s (Martyn 2003). In fact, individuals can tolerate substantial amounts of Alzheimer’s pathology in their brains without experiencing dementia, suggesting it may be not only the plaques that cause neurodegeneration and dementia in AD, but other disease related processes such as the neuroinflammation (Bronzuoli et al. 2016). Chronic neuroinflammation is one of the common features in AD and it is one of the mechanisms that may intensify the development of Aβ pathology and significantly contribute to neurodegeneration. Accordingly, it is increasingly considered as a potential therapeutic target for AD.

In this project, the pathogenesis of AD is examined in the context of chronic inflammation by using the STZ C57BL/6 animal model for sporadic AD instead of a transgenic AD mouse model. Transgenic mice, carrying mutations in the gene for amyloid precursor protein (APP), are widely used as a model AD. However, these animals rather resemble the familial form of AD (fAD), accounting for only 5–10% of all AD cases. Therefore, in line with recent finding that insulin resistant brain state plays a critical role in early sporadic AD pathology (Craft et al. 2012; Craft and Watson 2004), a new, non-transgenic, animal model has been proposed as an experimental model of sporadic AD. ICV-STZ in rodents was shown to cause memory impairment and progressive cholinergic deficits, as well as forming Aβ-like aggregates and causing abnormal Tau hyperphosphorylation, resembling those in AD patients (Nazem et al. 2015). Using the ICV-STZ model, we show that MIF-deficiency (genetically or pharmacologically) attenuates proinflammatory cytokine production and improves cognitive behavior in mice.

Excess MIF has been documented in CSF of patients clinically diagnosed with amnestic MCI and mild AD (Popp et al. 2009), suggesting that MIF could play a role in the pathogenesis of AD at the pre-dementia and early dementia stages. Emerging evidence suggests that deficiency of MIF attenuates tau hyperphosphorylation (Li et al. 2015). In a recent report analyzing the most recent publicly available ‘omics’ data, including genomics, epigenomics, proteomics and metabolomics data, a ranking algorithm was developed to prioritize the anti-AD targets, which revealed CD33 and MIF as the strongest candidates (Zhang et al. 2016). MIF is an upstream regulator for other cytokines, thus reasonably inhibiting this molecule holds promise as protective treatment in neuroinflammation.

MIF’s contribution to the neurodegeneration in ICV-STZ model of AD seems to be at various levels. DNA damage is thought to be the prime activator in STZ driven neurodegeneration in this model, inducing cellular mechanism resulting in apoptosis, necrosis and parthanatos. Poly(ADP-Ribose) Polymerase-1 (PARP-1) deficient mice are protected from STZ induced diabetes (Pieper et al. 1999), suggesting this pathway is essentially involved in neurodegeneration caused by this molecule. Intracellular MIF is responsible for translocation of apoptosis-inducing factor (AIF) into the nucleus and subsequent DNA fragmentation, which is a crucial step in PARP-1 dependent parthanatos (Wang et al. 2016). This proposes the role of intracellular MIF molecule in initiating neurodegeneration in ICV-STZ model, and explaining the in vivo and in vitro observation with MIF-KO cells and animals, that we observed no upregulation in cytokine levels after stimulation.

We and others have demonstrated that by triggering an ongoing and chronic immune response, STZ interferes with hippocampal dependent learning in C57BL/6 mice (Nazem et al. 2015; Sankowski et al. 2019). The observed deficits in contextual fear memory may be related to neuroinflammation or neurodegeneration caused by STZ in the hippocampal region. Inhibition of MIF using ISO-1 had a protective effect, which was reflected in increased average time of freezing of these mice in contextual fear conditioning paradigm in comparison to Veh-IP injected ICV-STZ mice.

Cognitive improvements in ICV-STZ mice following ISO-1 administration may be related to MIF’s role as upstream modulator to enhance the production of different cytokines, the inhibition of MIF during in vitro experiments resulted in downregulating the production and release of proinflammatory proteins. Unfortunately, the question if ISO-1 acts only in the CNS or in the CNS and the periphery remains to be answered. In cell culture studies, we previously have shown that blocking MIF using ISO-1 substantially reduces Aβ-mediated neurotoxicity, suggesting that a direct effect on microglia is involved (Bacher et al. 2010).

Hyperphosphorylation of tau, regulated by host kinases, results in the self-assembly of paired helical filaments, leading to the formation of abnormal structures in the cell body of neurons, known as neurofibrillary tangles. Tau hyperphosphorylation was shown to be attenuated in MIF deficient mouse models of AD (Li et al. 2015). We measured CSF and plasma MIF levels in subjects with prodromal or mild dementia AD and healthy controls without AD pathology. CSF MIF levels were higher in subjects with AD supporting the hypothesis that MIF expression in the CNS is increased at early clinical disease stages. In addition, we observed moderate correlations of MIF CSF levels with the CSF ptau181 and tau levels of subjects with AD.

In line with evidence from cell culture and animal studies, our findings in humans confirms and extends the correlation between MIF from the initial stages of AD with both total tau (t-tau) and phosphorylated tau (p-tau). Correlations of MIF with tau and p-tau in the CSF have been reported in recent studies on inflammation markers in neurodegeneration and AD (Brosseron et al. 2018; Craig-Schapiro et al. 2011). Our data, in combination with findings of Li et al. (Li et al. 2015), strongly imply that in addition to restricting neuroinflammatory response, the inhibition of MIF can restrain the tau affiliated progression of AD.

## Conclusion

In summary, our in vitro experiments underscore the important role of MIF in the inflammatory response, as the inhibition of MIF resulted in down-regulation of proinflammatory cytokines, whereas the levels of the anti-inflammatory IL-10 remained unaffected. In animal experiments, we observed improvement in cognitive function. The human data support a fundamental role of MIF in the inflammatory response to AD, and suggesting MIF may hold therapeutic potential for slowing down the progression of the disease.

## Supplementary information

**Additional file 1.**

## Data Availability

Please contact author for data requests.

## Abbreviations

Aβ: Amyloid β
AD: Alzheimer’s disease
AIF: Apoptosis-inducing factor
AMP: Adenosinmonophosphat
AMPK: AMP-activated protein kinase
APOE: Apolipoprotein E
APP: Amyloid precursor protein
CSF: Cerebrospinal fluid
CT: Threshold Cycle
ERK: Extracellular signal regulated kinase
fAD: Familial form of AD
IACUC: Institutional animal care and use committee
ICV: Intracerebroventricular
IP: Intraperitoneal
LPS: Lipopolysaccharide
MCI: Mild cognitive impairment
MIF: Macrophage migration inhibitory factor
NFκB: Nuclear factor ‘kappa-light-chain-enhancer’ of activated B-cells
n.s.: Not significant
PARP-1: Poly(ADP-Ribose) Polymerase-1
PI3K: Phosphoinositid-3-Kinase
SEM: Standard error of the mean
STZ: Streptozotocin
TREM2: Triggering receptor expressed on myeloid cells 2
Veh: Vehicle
