# Supplementary figures and images for: Key role of MIF-related neuroinflammation in neurodegeneration and cognitive impairment in Alzheimer’s disease
Source: Mol Med. 2020 Apr 17;26:34. doi: 10.1186/s10020-020-00163-5 (PMC7164357; doi:10.1186/s10020-020-00163-5)

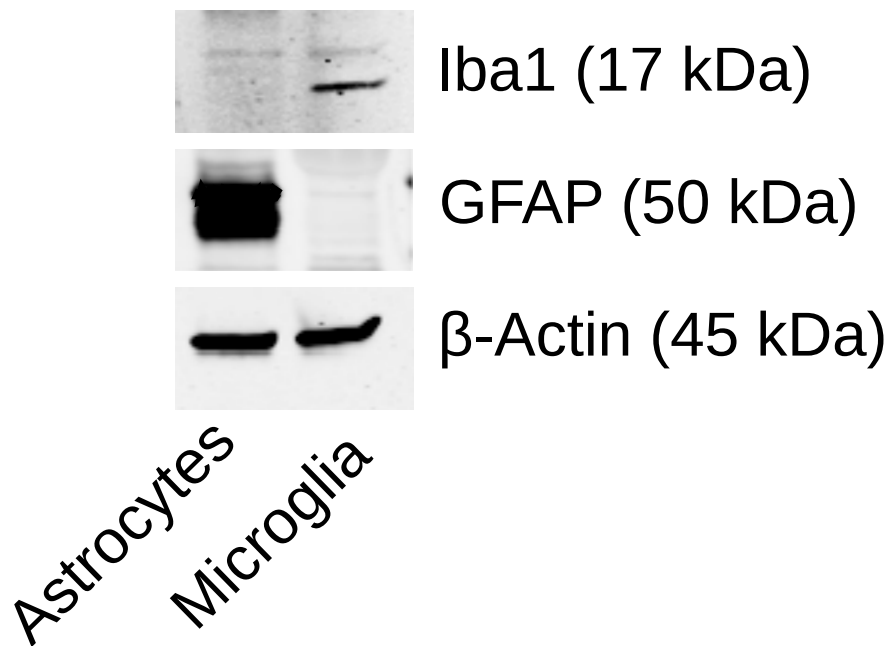

**Supplementary Figure 1**

Supplement: Supplementary file 1 — Additional file 1. [file 10020_2020_163_MOESM1_ESM.pdf]
